# Navigating the Storm: Managing Fetal and Neonatal Alloimmune Thrombocytopenia (FNAIT) in a High-Risk Pregnancy

**DOI:** 10.7759/cureus.49736

**Published:** 2023-11-30

**Authors:** Ekaterina Proskuriakova, Shikha Upreti, Joshua Wortsman, Bashar Alkhaurri, Jacob Rosendale, Mohammed Kassem, Pam Khosla

**Affiliations:** 1 Internal Medicine, Mount Sinai Hospital, Chicago, USA; 2 Internal Medicine, Ross University School of Medicine, St. Michael, BRB; 3 Internal Medicine, American University of the Caribbean School of Medicine, Cupe Coy, SXM; 4 Hematology and Oncology, Mount Sinai Hospital, Chicago, USA

**Keywords:** maternal alloimmunization, pregnancy, thrombocytopenia, case report, neonatal alloimmune thrombocytopenia

## Abstract

Fetal and neonatal alloimmune thrombocytopenia (FNAIT) is a rare, life-threatening condition causing significant thrombocytopenia and bleeding with the risk of developing intracerebral hemorrhage (ICH). It results from maternal immunizations against fetal platelet antigens. Here, we report a case of a pregnant patient at 30 weeks gestation who presented to the hospital with a low platelet count of 90 th/mm^3^ and was found to have anti-human platelet antigen (HPA) 1a, 2b antibodies. She was treated with a weekly infusion of IV immunoglobulins. However, her condition was complicated by the development of hemolysis, elevated liver enzymes, and low platelets (HELLP) syndrome, which was treated promptly with a platelet transfusion and intravenous magnesium. Even though the child had severe thrombocytopenia and its associated complications, there were no signs of post-delivery thrombocytopenia or any other adverse effects. This case report highlights the importance of the antenatal management of the FNAIT to prevent severe fetal complications, such as ICH.

## Introduction

Fetal and neonatal alloimmune thrombocytopenia (FNAIT) is a rare condition in pregnancy defined by maternal alloimmunization, resulting in the destruction of fetal platelets with a risk of severe bleeding [1]. The incidence of FNAIT is rare and occurs in 1/800-2000 live newborns [1,2]. 

About 85% of the cases of FNAIT are due to maternal-fetal incompatibility for human platelet antigen (HPA) 1a [3]. Maternally derived alloantibodies against paternally derived incompatible HPA cross the placenta and enter fetal circulation via active placental transport [4]. Occurrence of maternal alloimmunization in FNAIT might be attributed to various causes: previous feto-maternal hemorrhage during delivery or miscarriage involving leakage of fetal platelet content into the maternal circulation, prior maternal exposure to adult platelets, or release of syncytiotrophoblast microparticles (STMP) from the placenta [2]. As a result, alloimmunization leads to opsonization of fetal platelets, resulting in fetal/neonatal platelet destruction and thrombocytopenia [4]. The burden of thrombocytopenia is further enhanced as maternal anti-HPA-1a suppresses megakaryopoiesis via induction of cell death [5]. Moreover, maternal alloantibodies are capable of endothelium damage [1].

The clinical criteria for FNAIT consist of a platelet level under 100 × 10^9^/L in an affected infant or fetal intracranial hemorrhage (ICH) without other causes [6]. Most newborns with FNAIT present with mild clinical features such as petechiae, bruising, or asymptomatic thrombocytopenia [1]. However, 10-26% of mothers with anti-HPA-1a antibodies have been shown to have fetal/neonatal ICH. Furthermore, risk factors such as a fetus with an older sibling prior to pregnancy affected by antenatal ICH appear to be an independent risk for antenatal hemorrhage in the present pregnancy [2,7].

Currently, there is no routine prenatal screening to identify FNAIT. However, parents can be screened when FNAIT is suspected if there is a medical history of a previous neonate with unexplained thrombocytopenia, a prior fetus/neonate with ICH of uncertain etiology, or incidental finding of absent maternal HPA-1a [8]. Diagnosis can be achieved via flow cytometry, which detects platelet reactive antibodies, and solid phase assay, such as modified antigen capture enzyme-linked immunosorbent assay (ELISA MACE), can be used to detect specific glycoproteins the maternal antibody is targeting. If the father is heterozygous for incompatible platelet antigen, fetal platelet type can be determined from fetal DNA via amniocentesis or chorionic villus sampling (CVS) [3]. 

Here, we present a case of a 29-year-old female at 30 weeks gestation with a significant past medical history of immune thrombocytopenia (ITP) and past obstetric complication of thrombocytopenia who presented with decreased platelet counts and findings concerning for FNAIT.

## Case presentation

A 29-year-old female (G4P3A1 (had four pregnancies, three live births, and one miscarriage/abortion)), blood type B, with a past medical history of type two diabetes mellitus, ITP, and fetal and neonatal alloimmune thrombocytopenia presented to the maternal-fetal medicine (MFM) unit at 30 weeks gestation with a decreased platelet count of 90 th/mm^3^.

At the time of admission, she was asymptomatic. On review of systems, the patient denied dizziness, headache, shortness of breath, chest pain, and gastrointestinal or genitourinary symptoms. She endorsed having good fetal movements.

Her past medical history was significant for recurrent epistaxis. The patient’s family history was unremarkable. On examination, her vitals were within normal limits, with a temperature of 36.5 C, heart rate of 89 beats per minute, respiratory rate of 16 breaths per minute, blood pressure of 110/55 mmHg, and oxygen saturation of 98% on room air. Physical exam findings were unremarkable. She was alert, awake, and oriented, showing no apparent distress. Heart sounds had a regular rate and rhythm; lungs were clear to auscultation bilaterally; the abdomen was gravid, non-tender with no palpable masses, and the vaginal exam was deferred.

The patient reported that her youngest child, born three years ago, had a different father than her first two children and required transfusions of two units of platelets after being found to have thrombocytopenia shortly after birth with notable bruising on the scalp and face.

On admission, human platelet antigen testing for both the patient and her husband was done. The presence of maternal anti-HPA 1a and 2b antibodies was found in the patient. The father showed heterozygote incompatibility of HPA 1b, 3a, and 5b.

**Figure 1:**
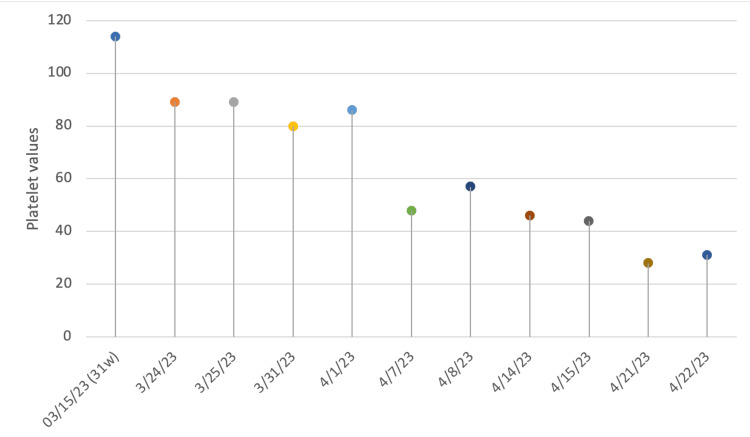
The trend of the platelets

Her laboratory results were significant for anemia of pregnancy with a hemoglobin value of 11.6 gm/dL, platelet count of 90 th/mm^3^, and elevated lipase of 117 U/L. Table 1 and Figure 1 represent her initial laboratory results on admission.

**Table 1:** Laboratory results on admission to the hospital Hgb: hemoglobin; MCV: mean corpuscular volume; MCHC: mean corpuscular hemoglobin concentration; RDW: red cell distribution width; MPV: mean platelet volume

	On admission	Range
WBC	7.7 th/mm^3^	4 – 11 th/mm^3^
RBC	3.93 mil/mm^3^	4.7 – 6.1 mil/mm^3^
Hgb	11.6 gm/dL	14.0 – 18.0 gm/dL (pregnant patient normal parameters: first trimester 11.6 - 13.9 gm/dL; second trimester 9.7 - 14.8 gm/dL; third trimester 9.5 -15 gm/dL)
MCV	85 Fl	78 – 100 Fl
MCHC	34.7%	32 – 36 %
RDW	14.7%	11.5 – 15.0 %
Plt Count	90 th/mm^3^	150 -400 th/mm^3^
MPV	11.6 Fl	7.5 – 12 Fl
Neutrophils	72.5%	2.0 – 7.5 %
Lymphocytes	18.1%	1.0 – 4.5%
Monocytes	6.5%	0.2 – 0.8 %
Eosinophils	2.7%	0.04% - 0.40%
Basophils	0.2%	<0.1%

Further work-up revealed the presence of HPA-1a alloantibodies against fetal platelets. The patient was referred from MFM for in-patient IV Ig (IVIG) infusion due to her clinical picture, her history of ITP, and her previous newborn presenting with findings concerning FNAIT.

The patient did not receive IV infusion treatment with IVIG until the 31st week of gestation. From 31 weeks of gestation until delivery, she was receiving IVIG 2 g/kg/week. Each weekly treatment was spread out over the course of two days with five-hour infusions per day. She was also taking 0.5 mg/kg/day prednisone until delivery. At each visit, a complete blood count was done to track her platelet levels. Before her treatment regimen started, platelets were at 114 th/mm^3^.

Figure 1 represents the changes in her platelet levels over the course of her treatment.

During this period, the patient persistently endorsed weekly worsening of her epistaxis in the mornings following the second round of treatment with IVIG. She did not endorse any other symptoms until day 39 of her treatment plan. On day 39, she presented with severe “10/10 pumping” chest pain and a headache with scotomas. She denied previously experiencing similar pain in the past. There was associated nausea and vomiting. On examination, her vitals were non-specific, with a temperature of 36.6 C, heart rate of 57 bpm, respiratory rate of 20 breaths/minute, blood pressure of 172/96, and oxygen saturation of 100% on room air. On physical exam, she was alert, awake, and oriented times three with anxious behavior; heart sounds displayed regular rate and rhythm without murmurs, lungs were clear to auscultation bilaterally with normal breath sounds, there was no respiratory distress nor accessory muscle use, the abdomen was gravid with no guarding or rebound tenderness and was soft to palpation, and there was no vaginal bleeding or abnormal vaginal discharge. Laboratory values were notable for sodium (Na) of 132 mEq/L (normal 135 to 145 mEq/L), platelet count of 31 th/mm^3^ (normal 150,000 - 450,000 th/mm^3^), hemoglobin of 11.4 gm/dL (normal 12 to 16 gm/dL), blood urea nitrogen (BUN) of 32 (normal 7 - 20 mg/dL), aspartate aminotransferase (AST) of 47 units/L (normal 10 to 36 units/L), and alanine aminotransferase (ALT) of 59 units/L (normal 7 - 55 units/L). MFM was consulted, and their evaluations were consistent with hemolysis, elevated liver enzymes, and low platelets (HELLP) syndrome.

IV magnesium 4 mg bolus was promptly given, followed by an hourly infusion of magnesium for seizure prophylaxis. She received a platelet transfusion and was urgently scheduled for delivery via cesarean section. In total, she received four units of platelets and three units of RBCs during and post-delivery.

Post-delivery, the neonate had no thrombocytopenia or any other complications. The patient and the newborn continued to be monitored to assess for any adverse clinical presentation caused by thrombocytopenia.

The patient was seen 21 days after discharge for follow-up. She and her newborn were both doing well. Her platelets increased to 202 th/mm^3^. She denied any bruising or epistaxis.

## Discussion

The 29-year-old (G4P3A1) patient with a significant past medical history of ITP and diabetes mellitus presented with labs significant for thrombocytopenia. Thrombocytopenia in pregnancy is defined as a platelet count less than 150 × 10^9^/L [9]. There was a suspicion of FNAIT during this pregnancy as the patient had a previous pregnancy complicated by neonatal thrombocytopenia, and the neonate was noted to have bruising on the scalp and face. 

FNAIT arises from a mismatch between the platelet-related antigens inherited from the mother and those from the father. Such incompatibility results in the production of alloantibodies against antigens specific to fetal platelets [2,10]. Maternal IgG crosses the placenta via neonatal Fc receptor, which subsequently opsonizes and destroys the fetal platelet [2]. The most common antibodies associated with FNAIT are specific for the HPA-1a epitope of β3 integrin. Other less common targeted antigens include HPA-5b and HPA-1. Of note, there are few cases of FNAIT in association with antibodies specific for HPA-2a/b, and it is reported that antibodies specific for HPA-2a/b are present in 20-40% of patients with ITP [11]. This patient’s HPA testing was significant for anti-HPA 1a 2b antibodies, and the paternal results revealed heterozygote incompatibility of HPA 1b, 3a, and 5b. 

The approach to management is guided to prevent severe complications of FNAIT, such as ICH and bleeding-related fetal/neonatal losses [12]. Patients are divided into categories according to the presence or absence of ICH in previous pregnancies. The presence of ICH in previous pregnancies is further divided according to the week of gestation in which the ICH was diagnosed [10]. The patient’s past obstetric history met the criteria for standard risk where there was no incidence of ICH, only thrombocytopenia. Such standard risk is initiated on 1 g/kg/week IVIG and 0.5 mg/kg/day prednisone or just 2 g/kg/week of IVIG at 20 weeks of gestation. At 32 weeks of gestation, IVIG dosage must be modified to 2 g/kg/week, and prednisone dosage remains the same at 0.5mg/kg/day [10,12]. The patient received treatment of 2 g/kg/week of IVIG and 0.5 mg/kg/day of prednisone until the time of delivery. 

Around 37-38 weeks of gestation, it is advised to have an elective cesarean delivery after fetal lung maturity is confirmed. However, vaginal delivery at 37-38 weeks of gestation can be considered if platelet count is higher than 100,000 mm^3^ [10]. The patient’s platelet count before treatment was documented at 114,000 mm^3^. The platelets were down-trending throughout her infusion treatment, with the lowest recorded at 28,000 mm^3^. Around 34 weeks and five days of gestation, the patient presented with signs and symptoms that were consistent with HELLP syndrome. 

Regarded as a variant of preeclampsia, HELLP syndrome is a severe complication of pregnancy with an incidence of 0.5%-0.9% [13]. The pathogenesis of this syndrome is unclear. There are different forms of HELLP syndrome. The presence of a triad defines the complete form: hemolysis, elevated liver enzyme, and low platelets. An incomplete or partial form may consist of one or two of the triad. Clinical presentation of this syndrome might include upper abdominal pain, headache, and visual symptoms [14]. Management of a patient diagnosed with HELLP syndrome takes into account gestational age. Gestational age beyond or at 34+0 weeks is treated via delivery of the fetus. If the previous criteria for gestational age are not met, a course of betamethasone is administered 48 hours before delivery. In addition, magnesium sulfate is administered for seizure prophylaxis [15]. The patient presented with platelet around 31,000 mm^3^, blood pressure of 186/103 mmHg, and mildly elevated liver enzyme; therefore, the patient was administered platelets, packed RBCs, a dose of betamethasone and IV magnesium before delivery via cesarean section. 

Post-delivery, the neonate’s labs were not significant for thrombocytopenia, and there was no complication such as ICH. On the last follow-up in the outpatient clinic, the patient’s platelets were recorded at 202,000 mm^3^. Both she and the newborn were doing well. 

## Conclusions

In conclusion, this case highlights the complexities of managing high-risk pregnancies in individuals with unique medical histories. The patient's history of type 2 diabetes mellitus and a previous pregnancy complicated by thrombocytopenia established a complex foundation for the current pregnancy. The primary focus was on the diagnosis and management of FNAIT, a rare condition with potential risks for the newborn due to maternal antibodies targeting fetal platelets. Despite the difficulties, the infant had a favorable outcome, showing a normal platelet count after birth. Both the mother and the newborn achieved positive results in follow-up outpatient monitoring, highlighting the significance of early identification and prompt intervention to alleviate potential adverse effects of FNAIT.
